# Effect of heat and mass transfer on the nanofluid of peristaltic flow in a ciliated tube

**DOI:** 10.1038/s41598-023-43029-6

**Published:** 2023-09-25

**Authors:** A. M. Abd-Alla, S. M. Abo-Dahab, M. A. Abdelhafez, Y. Elmhedy

**Affiliations:** 1 Mathematics Department, Faculty of Science, Sohag University, Sohag, Egypt; 2 Mathematics Department, Faculty of Science, South Valley University, Qena, Egypt

**Keywords:** Biophysics, Materials science, Mathematics and computing

## Abstract

The current work focuses attention on discussing the peristaltic flow of Rabinowitsch nanofluid through ciliated tube. This technical study analyzes heat and mass transfer effects on the flow of a peristaltic flow, incompressible, nanofluid via a ciliated tube. The governing non-linear partial differential equations representing the flow model are transmuted into linear ones by employing the appropriate non-dimensional parameters under the assumption of long wavelength and low Reynolds number. The flow is examined in wave frame of reference moving with the velocity $c$. The governing equations have been solved to determine velocity, temperature, concentration, the pressure gradient, pressure rise and the friction force. Using MATLAB R2023a software, a parametric analysis is performed, and the resulting data is represented graphically. The results indicate that the various emerging parameters of interest significantly affect the nanofluid properties within the tube. The present study enhances the comprehension of nanofluid dynamics in tube and offers valuable insights into the influence of heat and mass transfer in such setups. Convective heat transfer is found to be greater at the boundaries resulting in decreased temperature there.

## Introduction

Advances in fluids engineering and industrial sectors have aroused the interest of researchers in analysing mathematical models for non-Newtonian fluids. Non-Newtonian materials include slurries, coolants, lubricants, blood at low shear rates, ketchup, some paints, dirt, hygienic products, and many more. Several researchers have extensively studied the flow characteristics of such fluids due to their critical importance in the diverse fields of science and technology, including polymer solutions, viscoelastic suspensions, metal spinning, lubricants, plastics manufacturing, molten metal distillation, crystals, and food processing. Given its significance, a single constitutive model cannot encompass all non-Newtonian fluid aspects due to the diverse nature of fluid characteristics. The Peristaltic flow is a common method of fluid flow where flexible chambers are used to propel fluids through progressive waves of contraction and expansion. Akbar et al.^1^ studied the magnetohydrodynamics and convective heat transfer of nanofluids synthesized by three different shaped (brick, platelet and cylinder) silver nanoparticles in water. Narla et al.^2^ investigated the analysis of entropy generation in biomimetic electroosmotic nanofluid pumping through a curved channel with joule dissipation. Agoor et al.^3^ examined the binary powell- eyring nanofluid of peristaltic flow with heat transfer in a ciliated tube. Shit and Majee^4^ discussed a pulsatile MHD flow of blood signifying blood as a Newtonian fluid with temperature-dependent variable viscosity in an overlapping constricted tube under the influence of whole-body vibration. In a separate study, Shit and Majee^5^ examined the unsteady MHD flow of blood and thermal effect in an aneurysmatic artery using a finite difference approach. They documented that with increasing the magnitude of the Prandtl number, the Nusselt number enhances. Thermal radiation therapy is of significant importance in some medical procedures related to the cure of muscle spasm, myalgia (muscle pain), fibromyalgia and contracture. Maqbool et al.^6^ studied the effects of magnetic field and copper nanoparticles on the flow of tangent hyperbolic fluid through a ciliated tube. Tariq and Khan^7^ explained the behavior of second-grade dusty fluid flowing through a flexible tube whose walls are induced by the peristaltic movement. Ellahi et al.^8^ discussed the effects of heat and mass transfer on peristaltic flow in a non-uniform rectangular duct is studied under consideration of long wavelength and low Reynolds number. Nadeem et al.^9^ investigated the mathematical model for the peristaltic flow of nanofluid through eccentric tubes comprising porous medium. Shaheen and Nadeem^10^ analyzed the mathematical model of ciliary motion in an annulus is studied the effect of convective heat transfer and nanoparticle are taken into account. Iqbal et al.^11^ investigates the heat and mass transfer through curved channel with bi-convection. Main findings of the analysis discloses that the temperature of nanofluid decreases as radiation and viscosity varies. Abd-Alla et al.^12^ explained the effects of heat transfer and the endoscope on Jeffrey fluid peristaltic flow in tubes. Akhtar et al.^13^ construct polynomial scheme to find exact solution to the peristaltic flow of Casson fluid through elliptic duct. They focus their attention on the behavior of streamlines. Consideration of constant density force streamlines to be closed enough so that velocity get increased. Peristaltic flow of micropolar fluid through asymmetric channel with new type of interfacial thin film layer at the boundaries are studied by Mahmood et al.^14^. Tanveer et al.^15^ presented the analysis for peristaltic flow of Walter’s B fluid with internal heat generation. They applied numerical technique based on shooting method which express results in terms of interpolating function. Mansour and Abou-zeid^16^ studied the influence of heat and mass transfer the peristaltic flow of non-Newtonian Williamson fluid in the gap between concentric tubes. Awan et al.^17^ explained the numerical treatment for dynamics of second law analysis and magnetic induction effects on ciliary induced peristaltic transport of hybrid nanomaterial. Akbar and Butt^18^ investigated the concerns with the mechanical properties of a Rabinowitsch fluid model and the effects of thermal conductivity over it. In recent years, researchers have extensively focused on the peristaltic flow of Newtonian and non-Newtonian fluids (see for example^19–31^ and several references therein. In Refs.(^34–40^), peristaltic flow with new parameters with or without endoscope has been discussed.

Recently, significant interest has been developed in studying a peristaltic phenomenon due to its significant implications in biological sciences and biomedical engineering. In the current study, an investigation is carried out to inspect the peristaltic pattern of the nanofluid model in the presence of mass and heat phenomenon. The flow modelling is followed with the small Reynolds number hypothesis while the long wavelength is premised. The influence of various emerging physical parameters in the obtained solutions is observed. The obtained expressions are utilized to discuss the role of emerging parameters on the flow quantities. Numerical computations have been used to evaluate the expression for velocity, temperature, concentration, the gradient pressure, rise pressure and the friction force of various interesting parameters. Finally, the effect of various emerging parameters is discussed through graphs. Finally, the detailed computational results are compiled and discussed with the physical interpretation of our results. The graphical upshots for the velocity, temperature, concentration, the gradient pressure, rise pressure and the friction force are examined for influentrial parameters.

## Flow description

**Figure 1:**
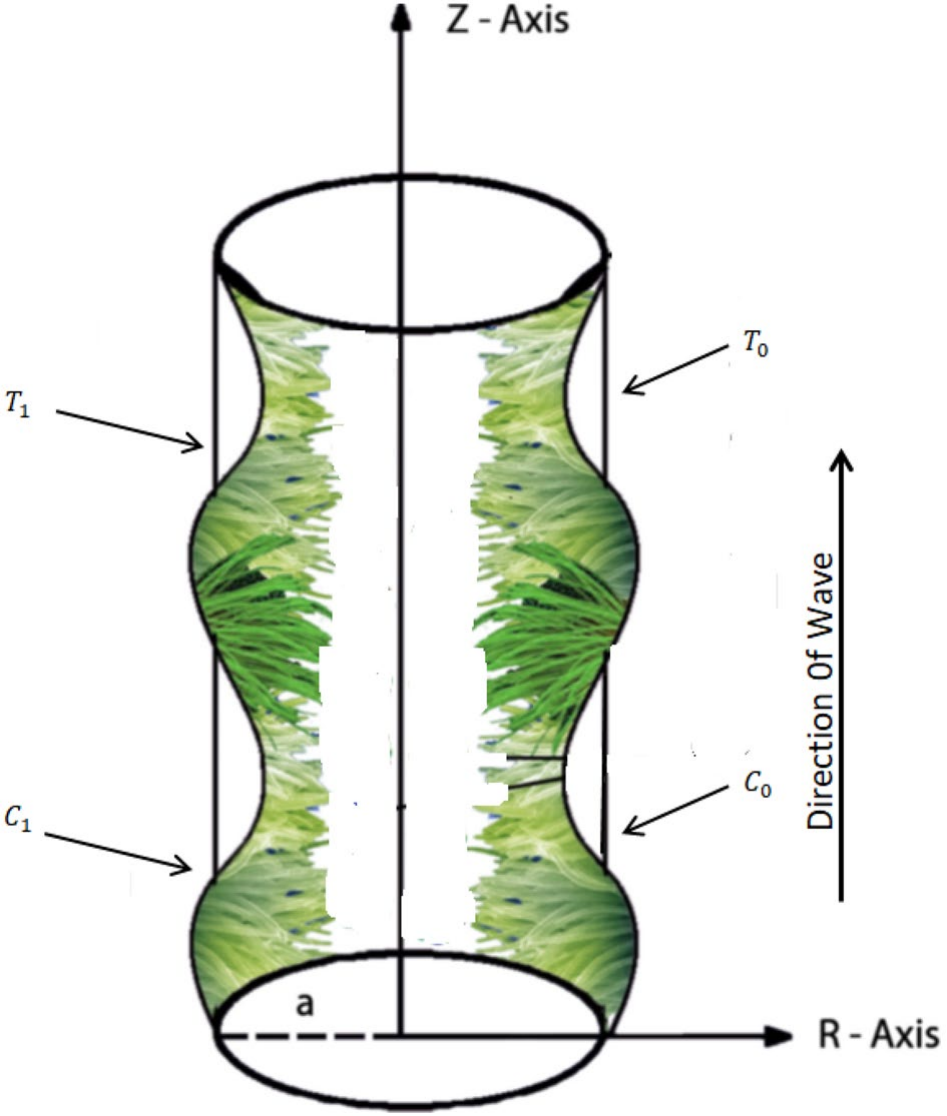
The schematic diagram of wall positions of tube when a peristaltic wave of slightly dilating amplitude propagates along it with velocity *c*.

The current analysis is performed to study the 2-dimensional peristaltic flow of Rabinowitsch nanofluid in an infinite tube with convective boundary condition. In addition, we also analyze the impact of nanofluid, thermal conductivity of fluid, the viscosity at constant concentration, thermal coefficient of nanofluid and heat source. For problem under consideration, geometry is exhibited in cylindrical coordinate system (r, θ, z) representing radial, azimuthal and axial coordinates respectively. Furthermore, the fluid flow is caused by the metachronal wave, resulting from uniform cilia beating whereas the temperature and concentration at the wall are $T_{0}$ and $C_{0}$ respectively (please, see Fig. 1).

## Formulation of the problem

For the peristaltic flow of an incompressible nanofluid with iron oxide nanoparticles in a uniform tube, using the cylindrical coordinate (*R*, *Z*) where *Z* the axis of the tube, while *R* the radius of it see Fig. 1. The flow is described in two coordinate systems one is fixed and the other, moving with speed *c*.

The equations of motion for the Rabinowitsch nanofluid model for the flow are^18,32,33^

1 $$\frac{\partial \overline{U} }{\partial \overline{R} }+\frac{\overline{U} }{\overline{R} }+\frac{\partial \overline{W} }{\partial \overline{Z} }=0.$$

The $r$ component of momentum equation:

2 $${\rho }_{nf}\left(\frac{\partial \overline{U} }{\partial \overline{t} }+\overline{U }\frac{\partial \overline{U} }{\partial \overline{R} }+\overline{W }\frac{\partial \overline{U} }{\partial \overline{Z} }\right)=-\frac{\partial \overline{P} }{\partial \overline{R} }+\frac{1}{\overline{R}}\frac{\partial }{\partial \overline{R} }\left(\overline{R }{\stackrel{-}{\overline{S}} }_{\overline{R }\overline{R} }\right)+\frac{\partial {\stackrel{-}{\overline{S}} }_{\overline{R }\overline{Z}} }{\partial \overline{Z} }-\frac{{\overline{S} }_{\overline{\theta }\overline{\theta }}}{\overline{R} }.$$

The $z$ component of momentum equation:

3 $${\rho }_{nf}\left(\frac{\partial \overline{W} }{\partial \overline{t} }+\overline{U }\frac{\partial \overline{W} }{\partial \overline{R} }+\overline{W }\frac{\partial \overline{W} }{\partial \overline{Z} }\right)=-\frac{\partial \overline{P} }{\partial \overline{Z} }+\frac{1}{\overline{R}}\frac{\partial }{\partial \overline{R} }\left(\overline{R }{\stackrel{-}{\overline{S}} }_{\overline{R }\overline{Z} }\right)+\frac{\partial {\stackrel{-}{\overline{S}} }_{\overline{Z }\overline{Z}} }{\partial \overline{Z} }+{\left(\rho \beta \right)}_{nf}g\left(\overline{T }-\overline{{T }_{0}}\right)+ {\left(\rho \beta \right)}_{nf}g\left(\overline{C }-{C}_{0}\right).$$

The energy equation:

4 $${\left(\rho {c}_{p}\right)}_{nf}\left(\frac{\partial \overline{T} }{\partial \overline{t} }+\overline{U }\frac{\partial \overline{T} }{\partial \overline{R} }+\overline{W }\frac{\partial \overline{T} }{\partial \overline{Z} }\right)={K}_{nf}\left(\frac{1}{\overline{R}}\frac{\partial }{\partial \overline{R} }\left(\overline{R }\frac{\partial \overline{T} }{\partial \overline{R} }\right)+\frac{{\partial }^{2}\overline{T} }{\partial \overline{{Z }^{2}}}\right)+{Q}_{0}.$$

The concentration equation:

5 $$\left(\frac{\partial \overline{C} }{\partial \overline{t} }+\overline{U }\frac{\partial \overline{C} }{\partial \overline{R} }+\overline{W }\frac{\partial \overline{C} }{\partial \overline{Z} }\right)={D}_{m}\left(\frac{{\partial }^{2}\overline{\complement } }{\partial {\overline{R} }^{2}}+\frac{1}{\overline{R} }\frac{\partial \overline{\complement } }{\partial \overline{R} }+\frac{{\partial }^{2}\overline{C} }{\partial {\overline{Z} }^{2}}\right)+\frac{{D}_{m}{K}_{T}}{{T}_{m}}\left(\frac{{\partial }^{2}\overline{T} }{\partial {\overline{R} }^{2}}+\frac{1}{\overline{R} }\frac{\partial \overline{T} }{\partial \overline{R} }+\frac{{\partial }^{2}\overline{T} }{\partial {\overline{Z} }^{2}}\right).$$

The constitutive equation for the extra stress tensor $\overline{S }$ is defined as follows:

6 $$\overline{S }=\frac{\mu }{1+{\lambda }_{1}}\left(\overline{\dot{\gamma } }+{\lambda }_{2}\overline{\ddot{\gamma } }\right).$$

The flow is obtained by a sinusoidal wave train which move with a constant speed *c* along the wall of the tube, wall equation of the tube in the fixed system is given by:

7 $$\overline{R }=H\left(\overline{Z },t\right)=a+a\varepsilon \mathrm{sin}\frac{2 \pi }{\lambda }\left(\overline{Z }-ct\right).$$

The equation of the cilia tips given mathematically in the form.

8 $$\overline{Z }={Z}_{0}+\alpha \varepsilon a \mathrm{sin}\frac{2 \pi }{\lambda }\left(\overline{Z }-ct\right).$$

The transformations between the two coordinate systems are:

9 $$\overline{r }=\overline{R } , \overline{z }=\overline{Z }-ct, \overline{u }=\overline{U } , \overline{w }=\overline{W }-c, \overline{p }=\stackrel{-}{P,}$$

where $(\overline{u },\overline{w })$ and $(\overline{U },\overline{W })$ represent the velocity components of the moving and fixed frame.

The boundary conditions are given by

$$\frac{\partial \overline{W} }{\partial \overline{R} }=0, \frac{\partial \overline{T} }{\partial \overline{R} }=0, \frac{\partial \overline{C} }{\partial \overline{R} }=0, at \overline{R }=0$$

10 $$\overline{W }=\frac{-2\pi \varepsilon a\alpha c}{\lambda }\mathrm{cos}\frac{2 \pi }{\lambda }\left(\overline{Z }-ct\right) , \overline{T }={T}_{0}, \overline{C }={C}_{0}, at \overline{R }=H.$$

The thermo-physical properties can be written as:

$$\rho_{nf} = (1 - \phi )\rho_{f} + \phi \rho_{s} ,$$

$$(\rho \beta )_{nf} = (1 - \phi )(\rho \beta )_{f} + \phi (\rho \beta )_{s} ,$$

$$\begin{gathered} (\rho c_{p} )_{nf} = (1 - \phi )(\rho c_{p} )_{f} + \phi (\rho c_{p} )_{s} , \hfill \\ \hfill \\ \end{gathered}$$

10a $$\frac{{K_{nf} }}{{K_{f} }} = \frac{{(K_{s} + 2K_{f} ) - 2\phi (K_{f} - K_{s} )}}{{(K_{s} + 2K_{f} ) + \phi (K_{f} - K_{s} )}}.$$

The dimensionless parameter is given as follows:

11 $$r=\frac{\overline{r}}{a }, z=\frac{\overline{z}}{\lambda }, u=\frac{\overline{u}}{c \delta }, w=\frac{\overline{w}}{c }, Re=\frac{{\rho }_{f}a c}{{\mu }_{f}}, h=\frac{H}{a}=1+\varepsilon \mathrm{cos}2 \pi z, {S}_{ij}=\frac{a}{{\mu }_{f}c}{\overline{S} }_{ij}, p=\frac{{a}^{2}\overline{p}}{{\mu }_{f}c \lambda }, Q=\frac{{Q}_{0}{a}^{2}}{\left({T}_{1}-{T}_{0}\right){K}_{f}}, \delta =\frac{a}{\lambda }, t=\frac{c \overline{t}}{\lambda }, \theta =\frac{\overline{T }-{T}_{1}}{{T}_{1}-{T}_{0}},\Theta =\frac{}{} , {G}_{r}=\frac{g {\rho }_{f}{B}_{f}{a}^{2}\left({T}_{1}-{T}_{0}\right)}{ c}, Br=\frac{\rho g {\alpha }_{c }\left(-\right)}{ c}, Sr=\frac{{\rho }_{f}{ D}_{m} {K}_{T} ({T}_{1}-{T}_{0})}{\left({C}_{1}-{C}_{0}\right)}, Sc=\frac{{\mu }_{f}}{{ D}_{m}{\rho }_{f}} .$$

## Solution of the problem

In view of the above transformations (9) and non-dimensional variables (11), Eqs. (1, 2, 3, 4 and 5) are reduced to the following forms:

12 $$Re {\delta }^{2}\left(u \frac{\partial u}{\partial r}+\left(w+1\right)\frac{\partial u}{\partial z}\right)=-\frac{\partial p}{\partial r}+\delta \frac{1}{r}\frac{\partial }{\partial r}\left(r {S}_{rr}\right)+{\delta }^{2}\frac{\partial {S}_{rz}}{\partial z}-\delta \frac{{S}_{\theta \theta }}{r},$$

13 $$Re \delta \left(u\frac{\partial w}{\partial r}+\left(w+1\right)\frac{\partial w}{\partial z}\right)=-\frac{\partial p}{\partial z}+\frac{1}{r}\frac{\partial }{\partial r}\left(r{S}_{rz}\right)+\delta \frac{\partial {S}_{zz}}{\partial z}+\frac{{\left(\rho \beta \right)}_{nf}}{{\left(\rho \beta \right)}_{f}}Gr \theta +\frac{{\left(\rho \beta \right)}_{nf}}{\rho {\alpha }_{c}}Br\Theta ,$$

14 $${\left(\rho {c}_{p}\right)}_{nf} c a \delta \left(u\frac{\partial \theta }{\partial r}+\left(w+1\right)\frac{\partial \theta }{\partial z}\right)={K}_{nf}\left(\frac{1}{r}\frac{\partial }{\partial r}\left(r\frac{\partial \theta }{\partial r}\right)+{\delta }^{2}\frac{{\partial }^{2}\theta }{\partial {z}^{2}}\right)+Q{K}_{f,}$$

15 $$Re \delta \left(u\frac{\partial\Theta }{\partial r}+\left(w+1\right)\frac{\partial\Theta }{\partial z}\right)=\frac{1}{Sc}\left(\frac{{\partial }^{2}\Theta }{\partial {r}^{2}}+\frac{1}{r}\frac{\partial\Theta }{\partial r}+{\delta }^{2}\frac{{\partial }^{2}\Theta }{\partial {z}^{2}}\right)+Sr\left(\frac{{\partial }^{2}\theta }{\partial {r}^{2}}+\frac{1}{r}\frac{\partial \theta }{\partial r}+{\delta }^{2}\frac{{\partial }^{2}\theta }{\partial {z}^{2}}\right).$$

and the boundary conditions in dimensionless form are:

$$\frac{\partial w}{\partial r}=0, \frac{\partial \theta }{\partial r}=0, \frac{\partial\Theta }{\partial r}=0, at r=0$$

16 $$w=-1, \theta =1,\Theta =1, at r=h.$$

Applying the approximation long—wavelength $\lambda > > 1,$ small Renolds number *Re* ≪ 1 and neglecting the wave number $\delta$. Since Reynolds numbers are typically very low (*Re* ≪ 1) in flow in small diameter tubules, analysis can be completed by approximating the inertia-free flow. Additionally, if the tube length is finite and equal to an integral number of wavelengths along with a constant pressure difference at the ends of the tube, the flow may be steady in the wave frame of reference. The governing Eqs. (12), (13), (14) and (15) in this situation, which make use of the long wavelength approximation, can be reduced to the following:

17 $$\frac{\partial p}{\partial r}=0,$$

18 $$-\frac{\partial p}{\partial z}+\frac{1}{r}\frac{\partial }{\partial r}\left(r{S}_{rz}\right)+\frac{{\left(\rho \beta \right)}_{nf}}{{\left(\rho \beta \right)}_{f}}Gr \theta +\frac{{\left(\rho \beta \right)}_{nf}}{\rho {\alpha }_{c}}Br\Theta =0 ,$$

19 $${K}_{nf}\left(\frac{1}{r}\frac{\partial }{\partial r}\left(r\frac{\partial \theta }{\partial r}\right)+{\delta }^{2}\frac{{\partial }^{2}\theta }{\partial {z}^{2}}\right)+Q{K}_{f}=0,$$

20 $$\left(\frac{{\partial }^{2}\Theta }{\partial {r}^{2}}+\frac{1}{r}\frac{\partial\Theta }{\partial r}\right)+SrSc\left(\frac{{\partial }^{2}\theta }{\partial {r}^{2}}+\frac{1}{r}\frac{\partial \theta }{\partial r}\right)=0,$$

$${S}_{rr}=\frac{2\delta }{1+{\lambda }_{1}}\left[1+\frac{{\lambda }_{2}c \delta }{a}\left(u\frac{\partial }{\partial r}+w\frac{\partial }{\partial z}\right)\right]\frac{\partial u}{\partial r},$$

21 $${S}_{rz}=\frac{1}{1+{\lambda }_{1}}\left[1+\frac{{\lambda }_{2}c \delta }{a}\left(u\frac{\partial }{\partial r}+w\frac{\partial }{\partial z}\right)\right]\left(\frac{\partial w}{\partial r}+{\delta }^{2}\frac{\partial u}{\partial z}\right),$$

$${S}_{zz}=\frac{2\delta }{1+{\lambda }_{1}}\left[1+\frac{{\lambda }_{2}c \delta }{a}\left(u\frac{\partial }{\partial r}+w\frac{\partial }{\partial z}\right)\right]\frac{\partial w}{\partial z},$$

$${S}_{\theta \theta }=\frac{2\delta }{1+{\lambda }_{1}}\left[1+\frac{{\lambda }_{2}c \delta }{a}\left(u\frac{\partial }{\partial r}+w\frac{\partial }{\partial z}\right)\right]\frac{u}{r}.$$

The solutions of velocity, temperature and concentration with subject to boundary conditions (16) are given by

22 $$\theta =1+\frac{}{4 {K}_{nf}} \left({h}^{2}-{r}^{2}\right) ,$$

23 $$\Theta =1+\frac{Sr Sc}{ 4}\frac{}{ {K}_{nf}}\left({r}^{2}-{h}^{2}\right),$$

24 $$w=\frac{{c}_{1} {r}^{4}-4{c}_{2}K {r}^{2}-4 {c}_{1}{h}^{2} {r}^{2}-4 {c}_{2}K {\lambda }_{1}{r}^{2}-4 {c}_{1} {h}^{2}{r}^{2}{\lambda }_{1}+{c}_{1}{ \lambda }_{1}{r}^{2} }{16 K}+{C}_{3},$$

The gradient of the pressure:

25 $$\frac{dp}{dz}=\frac{8 {c}_{1 }}{{h}^{2}\left({\lambda }_{1}+1\right)}-\left[\frac{{\left(\rho \beta \right)}_{nf} Gr}{{\left(\rho \beta \right)}_{f}}+\frac{{\left(\rho \beta \right)}_{nf} Br}{\rho {\alpha }_{c}}\right]-\frac{5 {c}_{1} {h}^{2} K}{6\left({\lambda }_{1}+1\right) }+\frac{{c}_{1}K {\lambda }_{1} }{4\left({\lambda }_{1}+1\right) }-\frac{{c}_{1}{h}^{2}K {\lambda }_{1}}{\left({\lambda }_{1}+1\right)}-\frac{8 F}{{h}^{4}\left({\lambda }_{1}+1\right)},$$

The volumetric flow rate:

26 $$F={\int }_{0}^{h}w r dr.$$

The rise of pressure:

27 $$\Delta {p}_{\lambda }={\int }_{0}^{1}\frac{dp}{dz} dz.$$

The force of friction:

28 $${F}_{\lambda }={\int }_{0}^{1}{h}^{2}\left(-\frac{dp}{dz}\right)dz,$$

where

$${c}_{1}=\frac{{\left(\rho \beta \right)}_{nf} Gr}{{\left(\rho \beta \right)}_{f}}-Sr Sc\frac{{\left(\rho \beta \right)}_{nf} Br}{\rho {\alpha }_{c}}$$

29 $${c}_{2}=\frac{{\left(\rho \beta \right)}_{nf} Gr}{{\left(\rho \beta \right)}_{f}}+\frac{{\left(\rho \beta \right)}_{nf} Br}{\rho {\alpha }_{c}},$$

$${C}_{3}=-1+\frac{{h}^{2} {c}_{2} \left(1+{\lambda }_{1}\right) }{4}+\frac{{c}_{1} Q {h}^{2}\left(1+{\lambda }_{1}\right) }{4 K}-\frac{{c}_{1} Q {h}^{4}\left(1+{\lambda }_{1}\right) }{16 K}.$$

## Results and discussion

**Figure 2:**
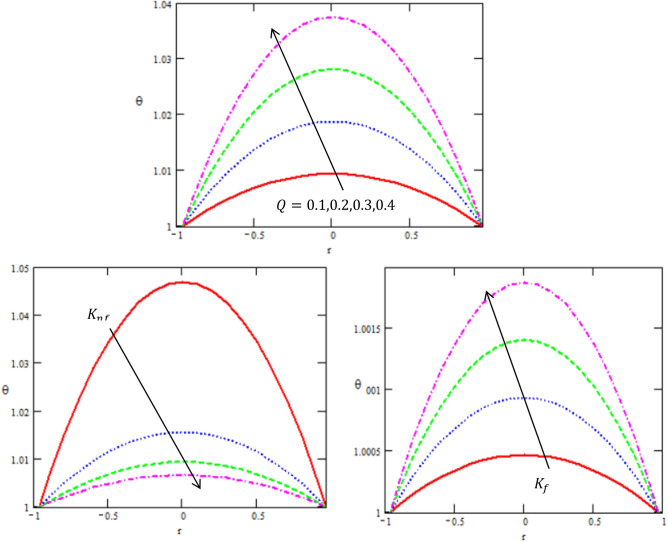
Variation between the temperature $\theta$ and the axial r with different values of $Q,{K}_{nf},{K}_{f}$.

**Figure 3:**
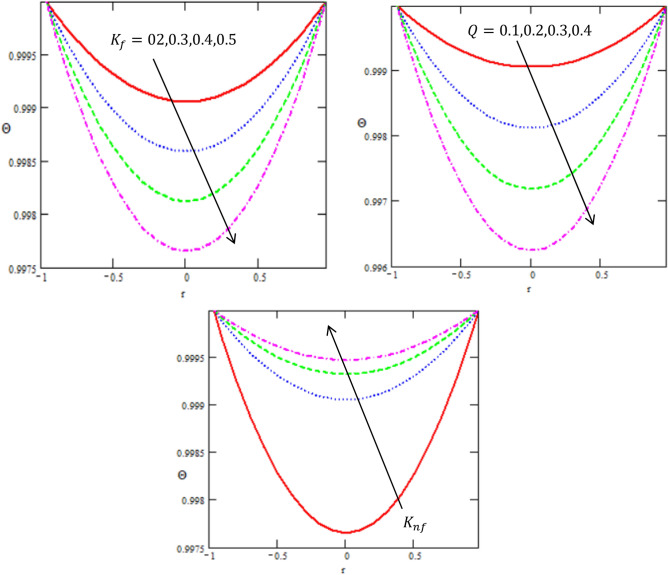
Variation between the concentration $\Theta$ and the axial-r with different values of $Q,{K}_{nf},{K}_{f}$.

**Figure 4:**
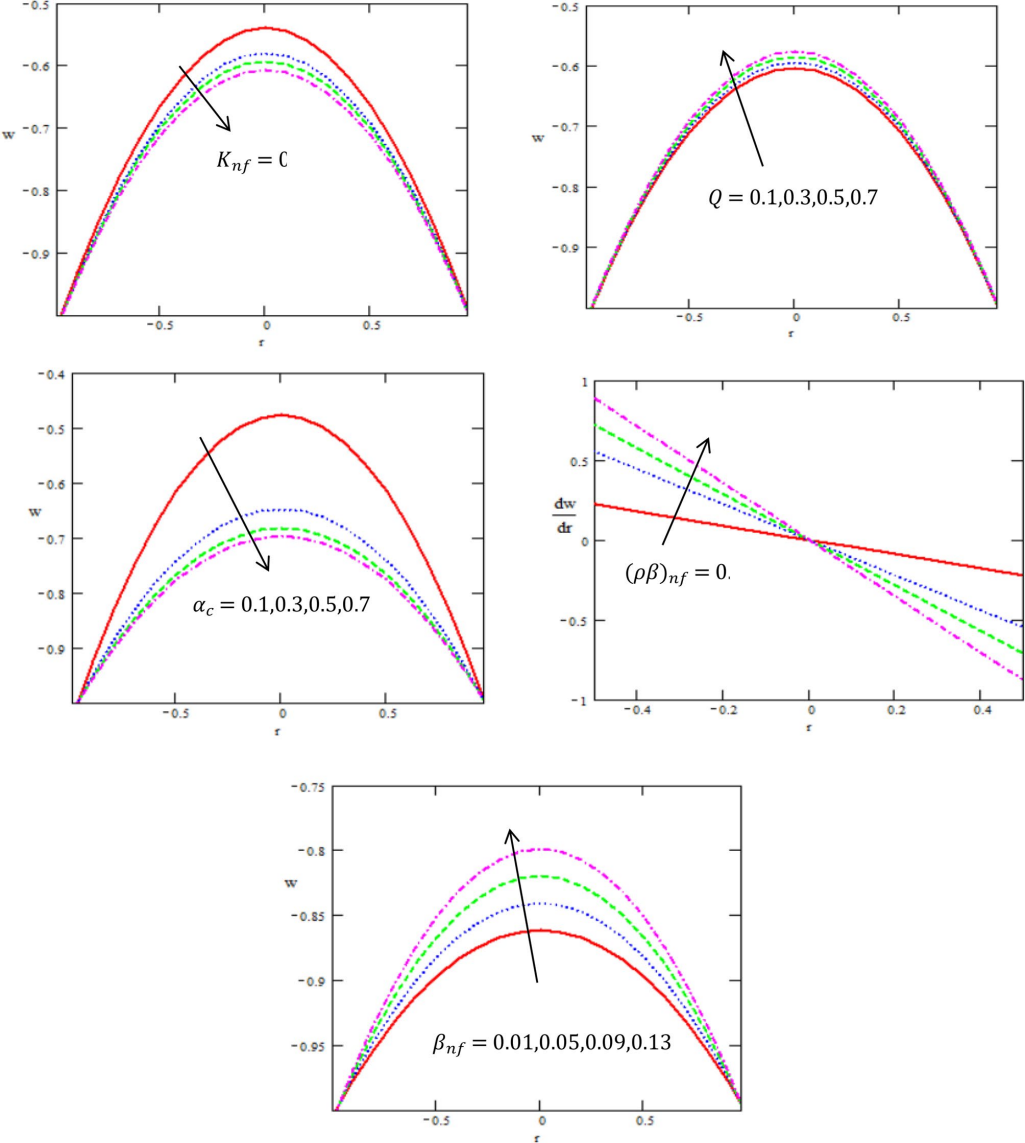
Variation between the velocity $w$ and the axial-r with different values of $Q,{K}_{nf}{\alpha }_{c},{\beta }_{nf}$.

**Figure 5:**
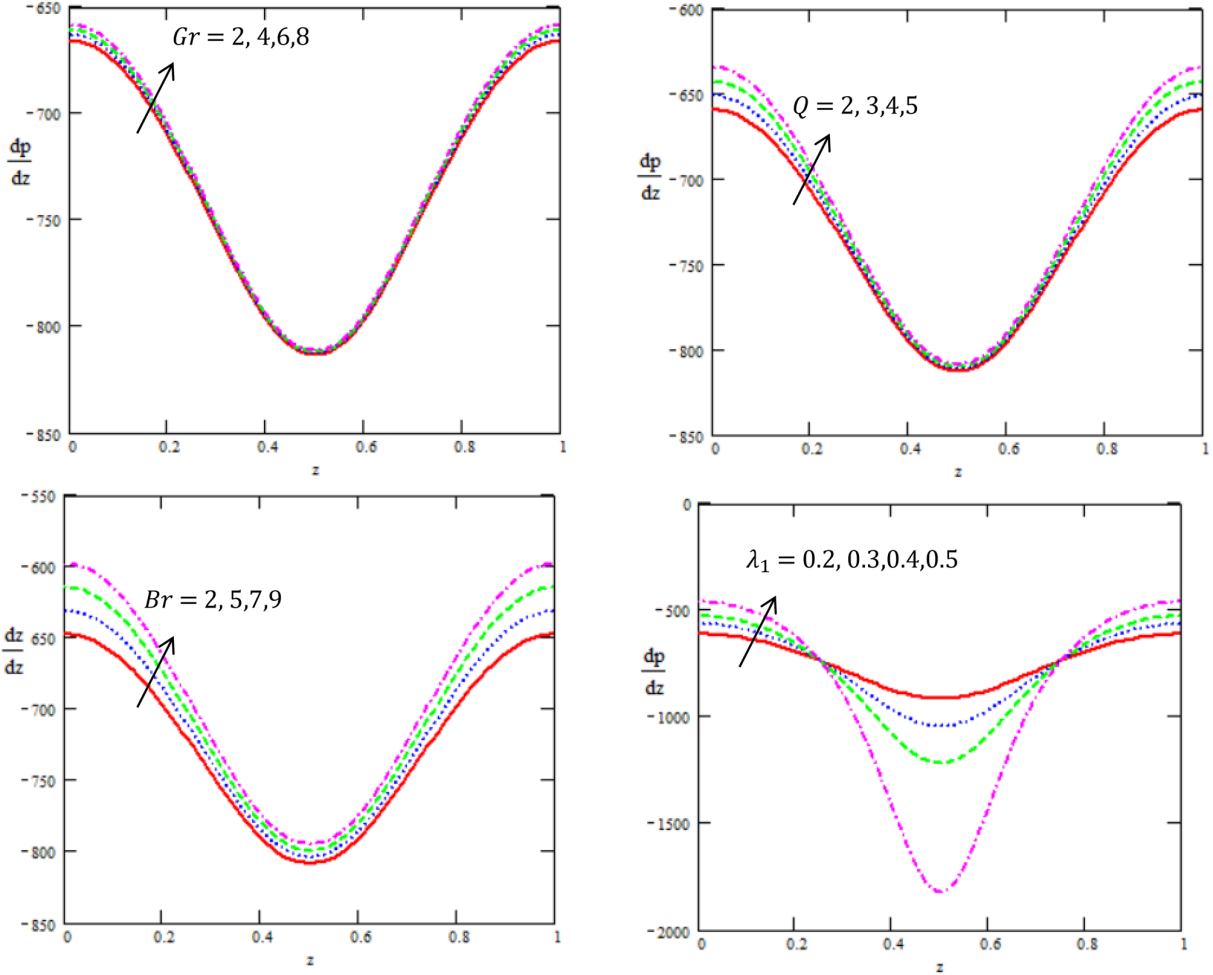
Variation between the gradient of pressure $\frac{dp}{{dz}}$ and the axial-z with different values of $Gr,\,Q,\,Br,\,\lambda_{1}$.

**Figure 6:**
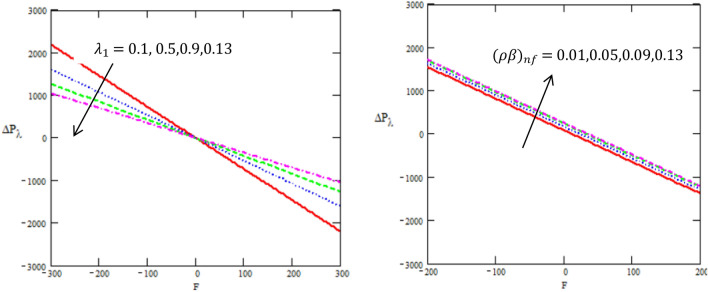
Variation between the rise of pressure $\Delta p_{\lambda }$ and the axial-F with different values of ${\lambda }_{1},{\beta }_{nf}$.

**Figure 7:**
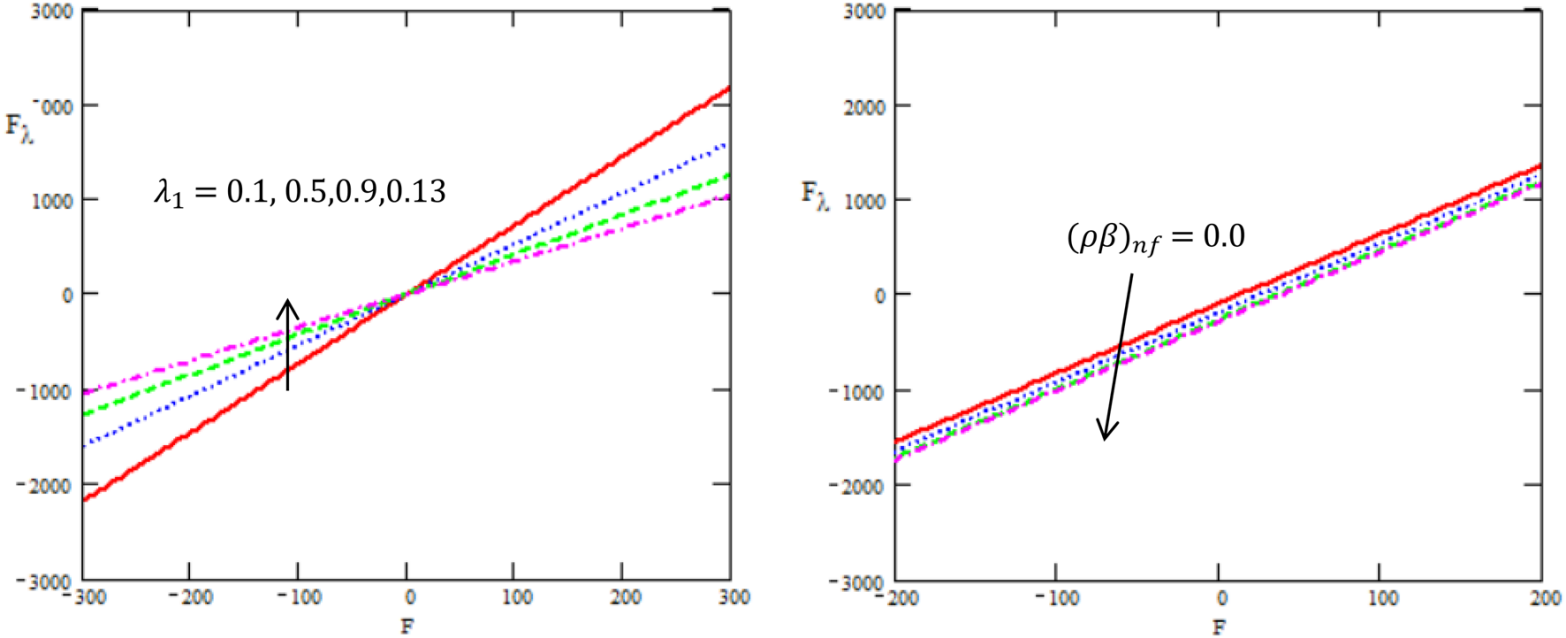
Variation between the force of friction $F_{\lambda }$ and the axial-F with different values of ${\lambda }_{1},{\beta }_{nf}$.

**Figure 8:**
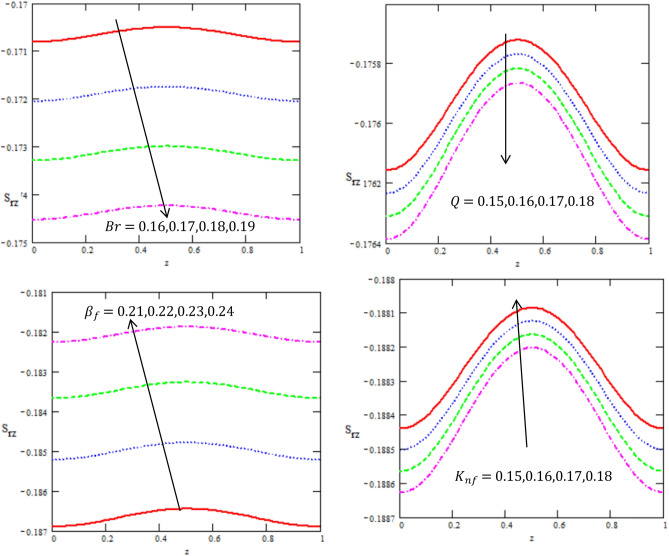
Variation between the shear stress $S_{rz}$ and the axis-z with different values of $Q,{K}_{nf},{\beta }_{f},Br$.

**Figure 9:**
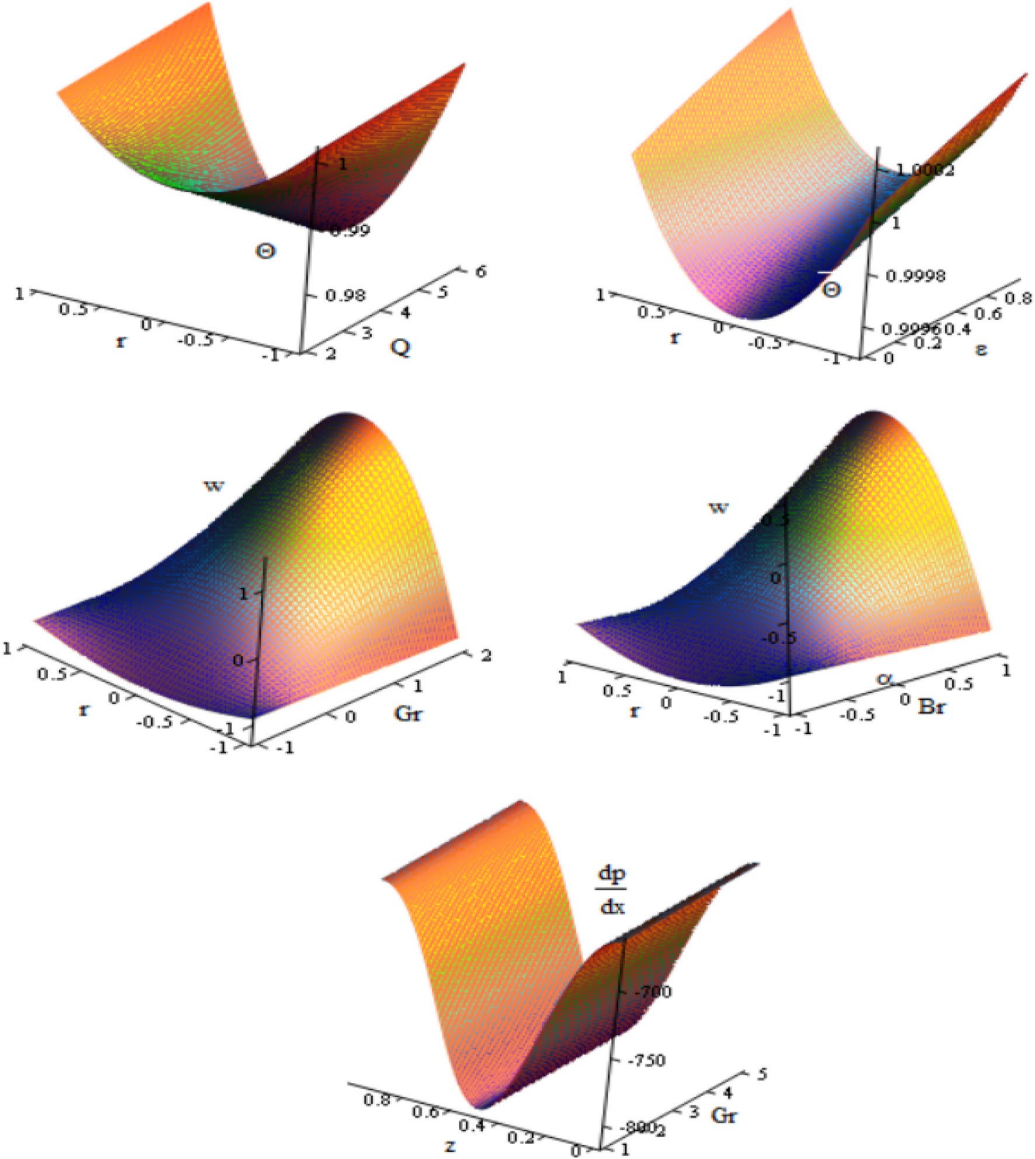
Discrepancies of the concentration $\Theta$ in 3D against $r$ and $z$ axis under the influence of $Q,\,\,\varepsilon ,$ velocity $w$ under the influence of $Gr,\,\,Br,$ pressure gradient $\frac{dp}{{dx}}$ under the influence of $Gr.$

This article discussed the Rabinowitsch Nanofluid of peristaltic flow with heat and mass transfer in a ciliated tube**.** The primary purpose of this section is to analyse the impact of pertinent parameters, including material parameters of Nanofluid, Nanofluid $K_{nf}$, thermal conductivity of fluid $k_{f}$, the viscosity at constant concentration $\alpha_{c}$, thermal coefficient of nanofluid $(\rho \beta )_{nf}$ and heat source $Q,$ on velocity ($w$), temperature (θ), concertation ($C$), pressure gradient $(\frac{dp}{{dx}})$, pressure rise $(\Delta p_{\lambda } )$, friction force $(F_{\lambda } )$ and shear stress $(S_{rz} )$. Graphs are drawn to analyse the effects of the relevant parameter mentioned above using the MATLAB 2023a programming language. For this purpose Figs. (2, 3, 4, 5, 6, 7, 8 and 9) are sketched to measure the features of all parameters. In particular, the variations of parameters are examined.

We disclose the influence of various parameters on temperature and heat transfer mechanism. Figure 2 shows the effect of thermal conductivity of nanofluid $K_{nf}$, thermal conductivity of fluid $k_{f}$ and heat source $Q.$ It is clear from the Fig. 3 that temperature surges as we increase the value of parameter. This is because of the fact that $K_{nf}$ > 0 indicates the heat generation. Which means temperature rises due to the internal friction of the nanofluid. elucidates the impact of convective heat transfer at the boundaries. It can be noticed from the temperature that for higher values of $Q$, temperature decreases. The reason behind this is the increased convective heat transfer at the boundaries which in turn results in decreased temperature, as well, we see an increasing manner in the temperature in the center of the tube. This result is in good agreement with the results obtained by Ellahi et al.^8^.

Mass transfer cannot be ignored while dealing with heat transfer during multiple industrial and physiological fluid transport. Therefore, current subsection deals with the influence of different parameters on concentration profile. Figure 3 depicts the impact of thermal conductivity of nanofluid $k_{nf}$, thermal conductivity of fluid $k_{f}$ and heat source $Q$ on concentration C. Mass concentration decreases for enhancing the values of $K_{f} ,\,\,Q,$ while it increases with increasing of $K_{nf} .$ Thus, increase in its value causes decrease in mass diffusion hence, resulting in mass accumulation. Thermal conductivity of nanofluid depicts the same behavior for increase in its concentration value. The behavior can be justified through the fact that increase in the value of $K_{nf}$ results in large concentration gradient which in turn moves more mass. Thus, causing increase in mass concentration. All these concentration graphs reveal a parabolic graphical outcome and the concentration is minimum in the centre of tube while it enhances toward the tube walls. This is in good agreement with what was obtained in clinical practice because the nutrients diffuse out of the blood vessels to neighbouring tissues^32^.

Figure 4 addresses the impact of related parameters on velocity profile $w$ of Rabinowitsch nanofluid flow through ciliated tube. Figure 4 provides the graphical plot of velocity for thermal conductivity of nanofluid $K_{nf}$, coefficient of the viscosity at constant concentration $\alpha_{c}$, thermal coefficient of nanofluid $(\rho \beta )_{nf}$ and heat source $Q.$ The increasing $K_{nf}$, $\alpha_{c}$ reveal a decline in the velocity profile, while it increases with increasing $(\rho \beta )_{nf} ,\,\,Q.$ Since, the flow rate is directly related to velocity, therefore causing this upsurge in velocity for both types of fluids. In the culmination of above discussion we can say that velocity exhibits parabolic behavior for Dilatant fluid. The velocity profile outcomes depict that the flow has a maximum speed in the centre and it eventually decreases towards the tube walls. For more authenticity, this result is in good agreement with the results obtained by Rafiq et al.^32^.

Figure 5 shows the axial pressure gradient expressed in terms of independent variable *z* is plotted for various corresponding parameters. Fluctuating behavior is shown by pressure gradient attaining its maximum at $0,\,\,1$, whereas approaching minimum at $0.5$, while it disclose the graphical outcomes of pressure gradient $\frac{dp}{{dz}}$ for certain physical parameters. Figure 5 shows that $\frac{dp}{{dz}}$ increases for increasing value of heat source Q and Brinkman number $Br$, while it decline in the interval $[0,\,0.2],$ as well, it increases in the interval $[0.2,\,0.8],$ like that, it increases in the interval $[0.8,\,1]$ for increasing values of Grashof number $Gr$ and ratio of relaxation to retardation times $\lambda_{1}$. This shows the existence of high level flow through the tube without the need of greater pressure gradient. Figure is prepared to analyze the impact of $Gr,\,Q,\,Br$ and $\lambda_{1}$ on $\frac{dp}{{dz}}$. It is clear from the Figure that pressure gradient simply decreases along the boundary of infinite tube. This result is in good agreement with the results obtained by Rafiq et al. et al.^32^.

Figure 6 is developed to illustrate how embedding parameters correspond to pressure rise $\Delta P_{\lambda }$ per mean flow rate in this regard. Non-Newtonian fluid characteristics can easily be described by nonlinear nature of these curve. Figure 6 presents a graphical solution of $\Delta P_{\lambda }$ for increasing ratio of relaxation to retardation times $\lambda_{1} ,$ thermal coefficient of nanofluid $(\rho \beta )_{nf}$ and a decline in the value of $\Delta P_{\lambda }$ is observed in the region $\Delta P_{\lambda } \succ 0$ while it increases in the region $\Delta P_{\lambda } \prec 0$. It is observed that the graphical result of $\Delta P_{\lambda }$ for incrementing $\lambda_{1}$ and an increase in the value of $\Delta P_{\lambda }$ is noted in the region $\Delta P_{\lambda } \prec 0$ while it declines in the region $\Delta P_{\lambda } \succ 0,$ while for increasing $(\rho \beta )_{nf}$ and an increase in the value of $\Delta P_{\lambda }$ in the regions $\Delta P_{\lambda } \prec 0,$ $\Delta P_{\lambda } \succ 0$. Peristalsis, which occurred as a result of pressure difference, causes flow rate to be positive in the zone of peristaltic pumping, whereas peristalsis of the tube boundaries produces a free-pumping region. This result is in good agreement with the results obtained by Ellahi et al.^8^.

Figure 7 indicate the disparities of the friction force $F_{\lambda }$ on tube with regards to the rate of volume flow $F$ for different values of the ratio of relaxation to retardation times $\lambda_{1} ,$ thermal coefficient of nanofluid $(\rho \beta )_{nf}$ in the peristaltic flow in a ciliated tube. In both figures, it is clear that the friction force in a ciliated tube has a non zero value only in a bounded region of space. It is observed that the friction force increases with increasing of ratio of relaxation to retardation times in the interval $[ - 300,0]$, while it decreases in the interval $[0,\,300]$, as well it decreases with increasing of the rate of thermal coefficient of nanofluid. On the other hand, these figures show that $F_{\lambda }$ have an opposite behavior compared to the pressure rise $\Delta p_{\lambda }$ versus the physical parameters.

Figure 8 displays the disparities of the value of axial tangential stress $s_{rz}$ with regards to the $z -$ axis, which it has oscillatory behavior which may be due to peristalsis in the whole range of the $z$-axis for different values of the Brinkman number $Br$, heat source $Q,$ The thermal expansion coefficient $\beta_{f}$ and thermal conductivity of nanofluid $K_{nf}$. In both figures, it is clear that the value of tangential stress has a non zero value only in a bounded region of space. It is observed that the shear stress decreases with increasing of $Br,\,\,\,Q,$ while it increases with increasing of $\beta_{f} ,\,\,K_{nf}$. It is noticed that the variation in shear stress at the tube wall surface with and without nanoparticles in the fluid and varying values of the non-Newtonian fluid parameter. It is important to note that the shear stress at the tube wall exerted by the streaming flow carrying nanoparticles is higher in magnitude than that of fluid without nanoparticles. Moreover the values of shear stress are larger in case of a peristaltic fluid when compared with Newtonian fluid.

Figure 9 shows the $3D$ schematics concern the concentration $\Theta ,$ axial velocity $w$ and the axial pressure gradient $\frac{dp}{{dz}}$ with regards to $r$ and $z$ axes in the presence of heat source $Q$, the heat source $Q,$ Grashof number $Gr$ and the cilia length $\varepsilon$. It is observed that the concentration decreases with increasing $Q,$ while it increasing with increasing of $\varepsilon ,$ axial velocity increases with increasing of $Gr,$ while it decreases with increasing of $\varepsilon ,$ the pressure gradient increases with increasing of $Gr$. We obtain for all physical quantities, the peristaltic flow in 3D overlapping and damping when $r$ and $z$ increase to reach the state of particle equilibrium. The vertical distance has more significant of the curves were obtained, most physical fields are moving in peristaltic flow.

## Conclusion

The Rabinowitsch nanofluid of peristaltic flow with heat and mass transfer in the ciliated tube is investigated**.** The present investigation utilized nanofluid model in a tube to analyse the peristaltic flow of non-Newtonian fluid in a ciliated tube with heat and mass transfer. A thorough understanding of peristaltic flow regulation and malfunction is possible by incorporating numerous effects that mirror natural events, especially in small arteries. These discoveries shed crucial new light on the properties of peristalsis during blood flow in human circulatory system. The knowledge gathered from this study could help diagnose and treat vascular diseases and promote scientific research. The following is a summary of the main conclusions drawn from the model:

1. Results obtained from the study indicate different flow behavior for the dilatant and nanofluid.
2. Thermal conductivity: This phenomenon causes the temperature to fall during peristalsis. This indicates that the nature of heat transfer within the system influences temperature fluctuations.
3. Coefficient of the viscosity at constant concentration: The model shows that fluctuating viscosity raises both velocity and temperature during peristalsis. This suggests that viscosity impacts the temperature and flow dynamics of blood.
4. The graphical solutions of velocity depict that the flow has a maximum speed in the centre and it eventually decreases towards the duct walls.
5. The temperature graphs reveal a parabolic graphical outcome and increasing manner in the temperature in the center of the tube.
6. The concentration graphs reveal a parabolic graphical outcome and the concentration is minimum in the centre of duct whereas it enhances toward the duct walls.
7. The results presented in this paper should prove useful for researchers in science and engineering, as well as for those working on the development of fluid mechanics.

Finally, one can concludes that the optimum state of the cases under consideration is applied various emerging parameters as affect the nanofluid properties within the tube because of its utilitarian aspects in dealing the peristaltic flow with heat and mass transfer.

## Data Availability

The datasets used and/or analyzed during the current study available from the corresponding author on reasonable request.

## List of symbols

c: Wave speed
$$C$$: Concertation
$$\left( {\frac{dp}{{dx}}} \right)$$: Pressure gradient
$$(\Delta p_{\lambda } )$$: Pressure rise
$(F_{\lambda } )$: Friction force and
$$(S_{rz} )$$: Shear stress
*R*: Radius
*Z*: Axis of the tube
${Z}_{0}$: Reference position of the cilia
$k_{nf}$: Thermal conductivity of nanofluid
$k_{f}$: Thermal conductivity of fluid
$$Q$$*Q*: Heat source
$\alpha$: Measure of the eccentricity of the elliptic motion of the cilia
$\alpha_{c}$: Coefficient of the viscosity at constant concentration
$$Br$$: Brinkman number
$(\rho \beta )_{nf}$: Thermal coefficient of nanofluid
$\beta_{f}$: The thermal expansion coefficient
$Gr$: Grash of number
$$\lambda$$: Wavelength
$\lambda_{1}$: Ratio of relaxation to retardation times
${\lambda }_{2}$: Retardation time and dots denote the differentiation with respect to time
$$\varepsilon$$: Cilia length
$\overline{S }$: Extra stress tensor
$$Sr$$: Soret number
$$Sc$$: Schmidt number
$${\text{Re}}$$: Reynolds number
$$Q$$: Heat source
$Q_{O}$: Heat generation coefficient
$$g$$: Gravity constant
$$\delta$$: Wavenumber
$$\overline{\dot{\gamma } }$$: Shear rate
${\overline{T} }_{0}$: Temperature of the inner tube
$\overline{U }$ and$\overline{V }$: Velocity components in radial and axial directions
$w$: Velocity profile of Rabinowitsch nanofluid
$a$: Radius of the tube
$\rho_{s} ,\,(\rho \beta )_{s} ,\,(\rho c_{p} )_{s}$: Density, the thermal expansion coefficient, the effective heat capacitance
$K_{s}$: Thermal conductivity of the solid particle
θ: Temperature
